# Work-related musculoskeletal disorders among dental staff in Armed Force Hospital in Dhahran, Saudi Arabia

**DOI:** 10.4314/ahs.v22i2.69

**Published:** 2022-06

**Authors:** Mashael Khaled Alzayani, Khalid F Salama, Mubashir Zafar

**Affiliations:** 1 Airbase Hospital, Dhahran, KSA; 2 Department of Public Health, College of Public Health, Imamm Abdul Rehman Bin Faisal university, Dammam, Saudi Arabia

**Keywords:** Dentists, Dental practitioner, Low Back Pain, Musculoskeletal Disorders, Saudi Arabia

## Abstract

**Background:**

Work related musculoskeletal disorders' (WMSDs) are the most important public health challenge among dental staff. The objective of study was to ascertain the prevalence and risk factors for work related musculoskeletal disorder among dental staff in Dhahran, Saudi Arabia.

**Methods:**

It is a cross sectional study and 130 dental staff with at least 1 year of working experience were random selected from Armed Forced Hospital. The self-administered validated and structured Standardized Nordic questionnaire was used. Logistic regression analysis was done to determine the association of risk factors with WMSD.

**Results:**

the results of the present study revealed that there was a high incidence of MSDs in neck, shoulder, and lower back pain among dental personnel (72.6%). The common risk factors which contributed to WMSD were more than 5 year of experience (AOR 1.19(1.03–2.82)), Saudi nationality (AOR 4.88 (1.27 – 18.72)), working more than 12 hours (AOR 3.115 (1.258 7.578)) and resident doctors (AOR 1.14 (1.02 – 1.94)).

**Conclusion:**

The study conclude work related MSD were common with high rate of incidence among dental staff . There is need to make a policy which will reduce the burden of WMSD among dental staff.

## Introduction

Work related musculoskeletal disorder (WMSDs) common public health problem.^1^ it is defined as soft-tissue injuries that cause by sustained or sudden repetitive movements, vibration, force, and awkward positions.^2^ It can affect the nerves, tendons, muscles, joints, and cartilage in limbs, neck and lower back.^2^ The common reason for musculoskeletal disorders due to awkward position during working in different occupational duties. The physical cause that related to such occupations has been recognized as a risk factor of WMSDs.^2^

According to WHO, approximately 59 million healthcare workers were suffering from WMSD. These workers were exposed to a wide range of occupational hazards. These hazards effects the physical, emotional and economic aspects of workers and their families.^3^ Many work related musculoskeletal disorders (WMSDs) were associated with ergonomics.^4^ Dental staff usually work in asymmetric and uncomfortable positions such as rotating the head laterally with the arms extended out of the body. The common cause of WMSD among dental staff are exhaust of joints, muscles, especially at shoulder, back and neck, which trigger a symptom as head and back pain.^5^

In a study which was conducted among dental professionals and result found that 85% of dental staff were sufferedfrom WMSD. The study result revealed that WMSD influence on the daily activities of dental staff and it forces some of the dental worker to change their dental setting. Factors associated with work-related pain are specialty of work, gender, age and duration of interaction with patients.^6^

In a previous study which was conducted in Malaysia, findings showed that 44% of dental staff were suffered back pain and most common among dental technicians (52.4%). The common risk factor associated with back pain were poor posture.^7^

In study which revealed high prevalence (58.7%) of work related musculoskeletal pain among dentist and higher rates of pain in the neck region (24%) followed by lower back region (20%) and upper back (14.7%). The associated risk factors were found to be number of working hours, number of cases treated per day, posture, and repetitive shoulder and hand movements (P<0.05).^8^

Very few researches were done in the Saudi Arabia about prevalence of musculoskeletal disorders associated to working environment among dental staff. There is no clear policy related to occupational health and safety among workers in Saudi Arabia. It reflects a lack of insight in relation to nature of work and others contributing risk factors. Therefore, this study aimed to determine the prevalence and risk factors of WMSDs among dental staff at airbase hospital, Dhahran, KSA.

## Methods

### Study setting, Study Design and Sampling technique

King Abdulaziz Air base Armed Forces Hospital in Dhahran City, Eastern Province of Saudi Arabia. Dental department Containing 29 operating dental room for different specialty that serve between 1000 to 1448 patients include booked appointments and walk-in per week. The study design was a cross-sectional study. The participants of this study were selected through simple random sampling. Take list of general physician, consultant, specialist and assistant technician dental staff from the administration department then assign the arbitrary number of each staff. Random number generator software was used to required sample size.

### Sample Size, Sampling Technique and Inclusion and exclusion criteria

Most of the participants were female. Married participants were constituted 81.5%. Saudi nationals were 64.6%. The proportion of bachelor's degree holder was 64.6%. Resident doctors were 20.8%. Majority (60%) of participants have 5–10 year of experience. The proportion of workers who work 8–12 hours were 96.9%.The sample size was estimated based on the reported work-related musculoskeletal disorders in dental staff which is 90.2%.^9^ A sample of 130 subjects will be required to obtain a 95% CI of ±5% precision around musculoskeletal disorders prevalence estimate of 90.2 %. All dental staff including general physician, consultant, specialist and para-medical staff such as Technician, nurses who is working in the Armed Forced Hospital were included in the study. Those who were working as a administrative staff were excluded. New dental staffs who were working less than one year in hospital were also excluded. Trainee dental professional were also excluded from the study.

### Study Variables and data collection tool

Dependent variable: the primary outcome of the study is musculoskeletal disorders among dental staff. Independent variable: include socio-demographic characteristics especially the age and gender. Other factors include: Marital status, education background, job title, years of experience and daily working hours. The study tool was used for this study is Standardised Nordic questionnaires.^10^ It is validated and structured questionnaire. The questionnaire divided into two sections. The first section is an about demographic data of dental staff while the second section is focused on analysis of musculoskeletal symptoms among dental staff including different regions of the body.

### Statistical analysis

Data was entered and analysed in IBM statistical package for social science software (SPSS) version 25. Descriptive Statistics will be used descriptions of the Demographic characteristics such as mean, standard deviation for numerical data and frequency and percentages for categorical data. To determine the various risk factors associated with WMSD, logistic regression models was used. Pearson correlation was used to determine the relationship between WMSD and risk factors. P-value 0.05 was significant.

### Ethical consideration

Ethical consent has been taken from research ethical committee and hospital directory in the study hospitals. After consent from the participants, objective of study was explained. All the aspects of the subjects were kept confidential and used only for the study purpose. The study was conducted after receiving IRB from imam Abdulrahman Bin Faisal University (IRB NO : UGS-2019-03-319) approval date 21/11/2019, and from Armed Force Hospital in Dharan(IRB Protocol No: AFHER-1RB-2020-002) IRB effective: Date January 9, 2020 .

## Results

The Mean and SD of participant age is 39.82±7.593 Table 1.

**Table 1:** Socio-Demographic Characteristics of Study Participants. (n=130)

Characteristics	Frequency n (%)
**Age (years) (Mean ±SD)** 25–40 >40	39.82±7.593 78 (60%) 52 (40%)
**Gender**	
Male	54 (41.5%)
Female	76 (58.5%)
**Marital Status**	
Single	24 (18.5%)
Married	106 (81.5%)
**Nationality**	
Saudi	84 (64.6%)
Non-Saudi	46 (35.4%)
**Education level**	
Technical certificate/Diploma	18 (13.8%)
Bachelor	84 (64.6%)
Master	10 (7.7%)
PhD	7 (5.4%)
Board	11 (8.5%)
**Job Title**	
Resident	27 (20.8%)
Specialist	24 (18.5%)
Consultant	11 (8.5%)
Nurse	20 (15.4%)
Technician	48 (36.9%)
**Working Experience**	
1–5 Years	18 (13.9%)
5–10 Years	78 (60%)
>10 Years	34 (26.2%)
**Daily working hours**	
8–12 hours	126 (96.9%)
>12 hours	4 (3.1%)

The Prevalence of musculoskeletal symptoms in the sample (n=130) were as low back pain 82.3%, neck 82.3%, Shoulder 75.4% and Elbow/hand 30.8%. Table 2.

**Table 2:** Prevalence of Musculoskeletal symptoms among the participants (ache, pain or discomfort)

Body Region		Frequency (n)	Proportion (%)
**Low Back**	Yes	107	82.3
	No	23	17.7
**Neck**	Yes	107	82.3
	No	23	17.7
**Shoulder**	Yes	98	75.4
	No	32	24.6
**Elbow / Hand**	Yes	40	30.8
	No	90	69.2
WMSDs*(Work **Related** **Musculoskeletal** **disorder)**	Yes	99	76.2
	No	30	23.8

The relationship between MSDs and baseline characteristics Table 3. The job title and year of experience are statistically significant with MSDs (p-value<0.05). All other values are related with baseline characteristics but no significant.

**Table 3:** Relationship between Work related Musculoskeletal disorder (MSDs) with baseline characteristics of study participants

Characteristics		WMSDs n (%)	p-value
**Sex**	Male	54 (41.5%)	0.230
	Female	76 (58.5%)	
**Nationality**	Saudi	84 (64.6%)	0.088
	Non-Saudi	46 (35.4%)	
**Educational** **level**	Technical certificate/Diploma	18 (13.8%)	0.473
	Bachelor	84 (64.6%)	
	Master	10 (7.7%)	
	PhD	7 (5.4%)	
	Board	11 (8.5%)	
**Job Title**	Resident	27 (20.8%)	0.001*
	Specialist	24 (18.5%)	
	Consultant	11 (8.5%)	
	Nurse	20 (15.4%)	
	Technician	48 (36.9%)	
**Year of** **Experience**	1–5 Years	18 (13.9%)	0.003*
	5–10 Years	78 (60%)	
	>10 Years	34 (26.2%)	
**Daily** **working** **hours**	8–12 hours	126 (96.9%)	0.956
	>12 hours	4 (3.1%)	

The association of MSDs Symptoms with the risk factors according to body region Table 4. In low back region, change job and work interference were associated with MSDs Symptoms. Similarly neck and shoulder region these predictors were associated with MSDs symptoms

**Table 4:** Association between the potential risk factors and MSDs symptoms in the participants' different body regions using multiple logistic regression

Body region	Predictors	AOR*	95% Confidence Interval (C.I)	p-value
Low back	Change jobs	0.54	(0.10–2.53)	0.403
	Work interference in home	8.50	(3.05–23.07)	0.001
Neck	Change jobs	1.13	4.13–293.41	0.001
	Work interference in home	10.06	(3.94–25.70)	0.001
Shoulder	change jobs	2.25	(1.21–5.47)	0.001
	Work interference in home	0.54	0.41–3.13	0.801

In the logistic regression analysis, a new variable was created and named: presence of any MSDs. Here, any participant who reported body pain was counted. The risk factors that were studied are shown in Tables 5; they revealed association between gender male and female, marital status, Nationality, education level, job title, year of experience and daily working hours.

**Table 5:** Association of WMSDs with Base line characteristics of study participants

Characteristics		MSDs Crude Odd Ratio (95% CI)	MSDs Adjusted Odd Ratio (95% CI
**Gender**	**Male**	1*	1*
	**Female**	1.68 (0.71 – 3.93)	1.66 (0.61 – 4.51)
**Marital Status**	**Single**	1.92 (0.69 – 5.37)	1.31 (0.33 – 5.24)
	**Married**	1*	1*
**Nationality**	**Saudi**	2.22 (0.87 – 5.66)	4.88 (1.27 – 18.72)
	**Non-Saudi**	1*	1*
**Education level**	**Technical** **certificate/Diploma**	0.76 (0.13 – 4.30)	0.49 (0.03 – 6.67)
	**Bachelor**	0.67(0.16–2.82)	0.46(0.04–4.51)
	**Master**	1.77(0.28–11.12)	1.57(0.09–6.90)
	**PhD**	2.00(0.27–14.78)	2.59(0.19–34.71)
	**Saudi Board**	1*	1*
**Job Title**	**Resident**	1.17(1.03 – 1.84)	1.14 (1.02 – 1.94)
	**Specialist**	1.86(1.27–5.10)	1.79(1.23–3.43)
	**Consultant**	1.48(1.09–2.54)	1.08(1.00–2.87)
	**Nurse**	1.11(1.01–1.94)	1.27(1.02–3.35)
	**Technician**	1*	1*
**Year of** **Experience**	**1–5 Years**	1*	1*
	**5–10 Years**	1.09(0.22–3.67)	1.19(1.03–2.82)
	**>10 Years**	4.44(1.08–18.27)	1.26(1.08–3.82)
**Daily working** **hours**	**8–12 hours**	1*	1*
	**>12 hours**	1.93 (0.94 – 9.35)	3.115 (1.258 7.578)

* 1:Reference category

Correlational analysis reveals that number of predictors of MSDs which are presented in Table 6. All predictors were positively correlated with MSDs except change jobs in neck region which is negatively correlated.

**Table 6:** Statistical correlates and predictors of MSDs

Body Region	Predictors	Correlation r (p-value)
**Back Pain**	**change jobs**	0.14 (0.102)
	**Work interference in home**	0.42 (0.001)
**Shoulder**	**change jobs**	0.07(0.374)
	**Work interference in home**	0.47(0.001)
**Neck**	**change jobs**	-0.27(0.001)
	**Work interference in home**	0.51(0.001)

## Discussion

This study is revealed that high rate of WMSD prevalence (76%) among dental staff. Musculoskeletal disorders (MSDs) are work related diseases and many factors can cause it.^1^ The most common factors are year of experience, education qualification and daily working hours.

In the present study, most of the participants were female. Married participants were constituted 81.5%. Saudi nationals were 64.6%. The proportion of bachelor's degree holder were 64.6%. Resident doctors were 20.8%.

The study found that age group from 20 to 40 years was high rate of prevalence WMSD which correlate with the study conducted in the Saudi Arabia 11.

Result found that female gender was common WMSD compared to male gender. This results was consistent with the other studies results. 9,11–13 The reasons for this issue are female have low muscle tone and low strength, hormonal factor which contributed to osteoporosis.^14^

Majority (60%) of participants have 5–10 year of experience. The proportion of workers who work 8–12 hours were 96.9%. However, those who have 25–40 years of age, male gender, Saudi nationals, and bachelor's degree holder were suffered WMSDs. WMSDs prevalence were high among resident doctor. The main reason for resident doctors, less working experience doctors suffered from WMSD because they have lack of experience and adaptation to the hospital environment.

The data of the present study are in accordance with study 15 that reported complaints of neck pain 54.4% and back pain in 73.5 % among dental staff. Dentists had relatively more neck (63.7%) and back (79.1%) pain as compared with neck (46.9%) and back (69.0%) pain among auxiliary staff (dental assistants, dental technician and dental hygienists). Similar finding was revealed by previous study 16 approximately 85% of subjects experienced WMSD-associated symptoms that commonly affected the regions of lower back (65.7%), ankle/foot (41.5%) and shoulders (29.0%).

Moreover, those study participants who have 5–10 year of experience were suffered high rate of WMSD compared to those have less than 5 year of experience. However, those study participants who have 8–12 hours working were suffered high rate of WMSD compared to those working >12 hours. The present study has found that high prevalence (76.2%) of WMSDs among dental staff which is consistent with the results of other study which was conducted in Saudi Arabia (59.2%)^17^, but lower incidence in Australia (87.2%)^18^, Lithuania (86.5%)^19^, and Turkey (94%)^20^. The prevalence of WMSDs among medical physicians was higher reports in previous studies (41.7%) in Iran 3 and Indian population (25.9%) 21, but lower prevalence in Nigeria (91.3%)^11^.

In the present study, the most frequently affected body sites were low back pain 82.3%, neck 82.3%, Shoulder 75.4% and Elbow/hand 30.8%. These areas may correspond to the overcapacity of the spine using during work. It means that dentists posture contributed for this problem. The prolonged static load because of sustained muscle activity in the sternocleidomastoid or trapezius muscles may be a primary etiological factor for neck pain among dentists.^22^

The common factor found that those working more than 12 hours who had high prevalence of WMSD this results were consistent with other study results 23–28.

The results found in this study that most common predictor which correlate with WMSD is change in job (r= -0.27). it means when person have WMSD, he changes the job which decrease the WMSD. This result was consistent with other studies results. (r=-0.39) 29, in the United Arab Emirates (r=-0.17) 30, Hail (r= -0.33) and Riyadh^12^ (r= -0.19).

## Limitation of the Study

The results of this study are limited by finding that can be biased based on multiple factors such as workload, number of patients treated in a single sitting, work environment, type of working (government sector, clinics, hospital) and work stress may influence the results. This is the cross sectional study which cannot temporality of causal factors.

## Conclusion

The study conclude that the prevalence of musculoskeletal disorders and symptoms is very high among dental staff. The law back, neck and shoulders are the most frequently affected anatomical sited for muscular disorders. Dentist must follow the basic health and safety principles and be aware of correct working postures. In addition, regular rest intermissions between the patients and regular medical examination can be very proactive strategy.
